# Cancer and immune response: The role of PD-1/PD-L1 checkpoint in laryngeal carcinoma. Preliminary results

**DOI:** 10.1007/s00405-024-08822-7

**Published:** 2024-07-28

**Authors:** Barbara Verro, Giuseppe Saraniti, Gaetano Ottoveggio, Carmelo Saraniti

**Affiliations:** 1 Unit of Otorhinolaryngology, Department of Biomedicine, Neuroscience and Advanced Diagnostic (BIND), University of Palermo, Palermo, Italy; 2 Unit of Anesthesia, Intensive Care, and Emergency, Department of Surgical, Oncological and Oral Science (Di.Chir.On.S.), University of Palermo, Palermo, Italy

**Keywords:** Laryngeal carcinoma, Biomarker, Prognosis, Treatment, Immunotherapy

## Abstract

**Purpose:**

The overall survival of laryngeal squamous cell carcinoma (LSCC) hasn’t changed significantly in the last decades, leading to a negative prognosis in advanced stages. So, the immunotherapy takes space with the inhibition of PD-1/PD-L1 checkpoint, involved in suppression of immune response.

**Methods:**

A prospective study was conducted on LSCC patients, selected according to strict criteria. The study was approved by the ethics committee of our Hospital. Parameters were: sex, age, smoking and alcohol habits, C-reactive protein (CRP) level in the serum, laryngeal subsite involved, differentiation-based histopathologic grading of tumor, neck node involvement, tumor stage, expression levels of PD-L1 (as Combined Positive Score – CPS). *P*-value < 0.05 was statistically significant.

**Results:**

58 patients were included: 31.03% were females, mean age 63.55±10.09. A statistically significant correlation between CPS and smoking habits and *N* stage was found. CRP resulted increased in 44.83% of patients with a statistically significant correlation with CPS. The most cases were glottic cancers (46.55%). 75.86% of tumors were moderately-differentiated, without correlation with CPS.

**Conclusion:**

PD-L1 expression levels are variables independent of sex, age, alcohol consumption, differentiation degree of LSCC. A statistically significant correlation between PD-L1 expression levels and smoking habits, neck node status and CRP was found. This last finding demonstrates the involvement of PD-1/PD-L1 checkpoint in immune response in case of cancer. However, these results need further studies to detect the best patients tailored for treatment with anti-PD-1/PD-L1 blocking antibodies.

## Introduction

Laryngeal squamous cell carcinoma (LSCC) is among the most prevalent head and neck cancers worldwide [1]. In the last years, despite the overall incidence of LSCC is decreased [2] and despite advancements in diagnostic tools, around 60% of LSCC are still diagnosed at advances stages [3] resulting in a declining 5-year survival rate [2]. Based on these assumptions, the main goal of oncology is to formulate a customized therapy in order to ensure the best prognosis to patient. So, in this context, the immunotherapy has emerged as a significant approach, particularly with the inhibition of PD-1/PD-L1 (programmed cell death protein – 1 / programmed cell death 1 ligand 1) checkpoint, which plays a crucial role in immune response suppression [4]. In 1992, Tasuku Honjo found that protein PD-1, expressed on T cells surface, brakes its activity. This discovering paved the way to the use of PD-1 as a target for therapy against cancer. PD-L1, expressed by tumor cells, binds to the receptor PD-1 on T cells, leading to inhibition of their anti-tumor role [5] and to induction and maintenance of immune tolerance within the tumor microenvironment. In particular, PD-1/PD-L1 axis inhibits activation, proliferation, survival and cytotoxic secretion of T cells within cancer cells [6].

Studies found that administration of anti-PD-1/PD-L1 blocking antibodies (called pembrolizumab) is effective in treating recurrent or metastatic head and neck squamous cell carcinoma (R/M-HNSCC) [7–9]. Actually, patients with R/M-HNSCC have a median overall survival (OS) ranging from 9 to 12 months [10]. So, this new therapeutic strategy may improve the prognosis of these patients, as reported by a recent phase 3 study (KEYNOTE-048) which demonstrated the best efficacy and therapeutic response of pembrolizumab compared to cetuximab with chemotherapy (5-fluorouracil and platinum) [11]. Based on the results of this clinical study KEYNOTE-048, in 2019 the Food and Drug Administration (FDA) approved pembrolizumab, that is the monoclonal antibody PD-1 (known with the brand name Keytruda), as a first-line therapeutic drug for patients with R/M-HNSCC. Moreover, FDA recommended pembrolizumab in combination with platinum and fluorouracil for patients with advanced HNSCC and in monotherapy for patients whose PD-L1 Combined Positive Score (CPS) expression is ≥ 1% [11]. Another drug that inhibits the binding of PD-L1 to both PD-1 and CD80 is Nivolumab, IgG4 monoclonal antibody; it is also approved by the FDA for the treatment of R/M-HNSCC [12].

However, to date, the literature is poor of studies about the role of PD-L1 in LSCC [13–16]: most of them focused on evaluating the possible correlation between PD-L1 expression level on LSCC tissue and systemic inflammatory state. Indeed, some studies assessed and demonstrated the correlation between PD-L1 expression levels and tumor-infiltrating lymphocytes (TIL) count, suggesting the role of PD-L1 as marker of anti-tumoral response by immune system [13, 17–20]. Other studies proved higher PD-L1 expression level in advanced LSCC [14, 16, 21]. A better prognosis was found in case of high expression levels of PD-L1 [17, 20, 21]. About smoking, studies reported controversial results: Yang et al. found a higher PD-L1 expression in non-smokers [14], while Kowalski et al. in smoker patients, regardless of cigarette number and duration of this habit [16].

Our study aims to assess the possible correlation between PD-L1 expression levels, and some clinical data of patients affected by LSCC that may help in the detection of a target population for immunotherapy with anti-PD-1/PD-L1 blocking antibodies.

## Materials and methods

A prospective study was conducted on patients affected by LSCC referring to our Ear Nose and Throat Unit at our University Hospital, from January 2022 to January 2024. The study was carried out in accordance with the Declaration of Helsinki and approved by the ethics committee of our University Hospital (approval number 05/2023). Enrolled patients signed the informed consent to participate in this study.

Patients were selected according to the following inclusion criteria: males and females, over the age of 18, with newly diagnosed LSCC (first diagnosis), neither persistence nor recurrence of LSCC, presence of data in the hospital database about the parameters to evaluate. Exclusion criteria were: pregnancy; insanity defense; other synchronous and/or previous extra-laryngeal tumors, non-squamous cell carcinoma of the larynx.

### PD-L1 immunohistochemical staining

Anti-PD-L1 mouse monoclonal clone 22C3 was used to detect the PD-1 protein according to the qualitative immunohistochemistry exam (PD-L1 IHC 22C3 pharmDx kit) [22, 23]. First, PD-L1 IHC 22C3 pharmDx and hematoxylin and eosin (H&E) staining of serial slices of the paraffin specimen are performed to ensure tissue preservation quality and to confirm the diagnosis of LSCC. First, deparaffinization, rehydration, and target retrieval are performed by using PT Link. Then, specimens are first incubated with Negative Control Reagent (NCR), then with a Mouse Linker, at the end with a Visualization Reagent. The chromogen addition leads to its enzymatic conversion that results a visible at the site of the antigen thanks to a chromogen enhancement reagent. Assessments are performed by using the light microscope. The best PD-L1 interpretation is achieved by using three serial tissue slides (H&E, PD-L1, and NCR stains): if the first H&E results acceptable, IHC staining of PD-L1, and NCR sections should be acceptable too. Quality controls included in each staining run are: PD-L1 IHC 22C3 pharmDx Control Cell Line Slide stained with the primary antibody; negative and positive in-house control tissues stained with the primary antibody too; sections of each specimen stained with Negative Control Reagent. At least 100 viable cancer cells must be in the PD-L1 stained slice in order to perform an adequate PD-L1 assessment. As written above, PD-L1 expression in laryngeal cancer is evaluated by using CPS, that is the number of PD-L1 staining cells, both tumor cells and stromal cells (macrophages, lymphocytes) divided by the total number of viable tumor cells, multiplied by 100 [18]. PD-L1 expression was classified as: negative (CPS < 1), intermediate (1 ≤ CPS < 20), and strong expression (CPS ≥ 20).

### Data analysis

The following parameters were collected: sex, age, smoking and alcohol habits, C-reactive protein (CRP) level in the serum upon the hospital admission (cut-off < 5 mg/L), laryngeal subsite involved and tumor extension (supraglottic, glottic, subglottic, transglottic, extralaryngeal), differentiation-based histopathologic grading of LSCC (G1 or well-differentiated, G2 or moderately-differentiated, G3 or poorly-differentiated), neck node involvement (yes, not or not known), expression levels of PD-L1 (referred to as CPS), tumor stage (according to the 8th Edition of the American Joint Committee on Cancer) [24].

Based on daily cigarettes consumption, the patients were classified as non-smokers, light-smokers if smoked fewer than 20 cigarettes a day, heavy-smokers if smoked more than 20 cigarettes a day. Based on daily alcohol consumption, the patients were divided into non-drinkers, light-drinkers or heavy-drinkers if drank less or more than 4 drinks a day, respectively. The assumption was that a drink corresponds to 14 g of ethanol [25].

Data were collected on spreadsheets using Microsoft Excel (16.83 version) and reported as numbers, percentages, mean ± standard deviation (SD). Statistical analysis was performed using Chi-squared tests with Yates’ correction and univariate analysis was performed to evaluate possible correlation between CPS and clinical data. Two-tailed *p*-value < 0.05 was considered statistically significant. GraphPad Software© was used for this statistical analysis. Spearman correlation matrix was used to measure the strength and direction of the association between variables. Multivariate Regression Analysis was performed considering CPS as the dependent variable and other clinical parameters as independent variables.

## Results

According to the selection criteria, a total of 58 consecutive naïve patients were included in the study: (18/58) 31.03% were females, the mean age was 63.55 ± 10.09 years, with a range from 43 to 83 years. Most of the patients (91.38%) underwent surgery (transoral laser microsurgery or open laryngectomy, both partial and total), while 6.89% received non-surgical therapy, and 1 patient opted out of treatment, either surgical or conservative one. The univariate analysis of the variable “age” showed that the mean and median age are very close, indicating a fairly symmetrical distribution. No statistically significant correlation was found between CPS value and sex (*p*-value 0.0523). However, most patients of both sexes expressed a CPS higher than 1 (44.44% of women and 75% of men). The univariate analysis showed that most of the patients (89.66%) smoked and almost half of the sample (41.38%) consumed alcohol. All drinker patients (100%) were used to drink more than 4 drinks per day (heavy-drinkers); no light drinkers were present in the sample. About smoking, most patients (79.32%) smoked more than 20 cigarettes per day (heavy-smokers). A statistically significant correlation between PD-L1 expression levels and smoking habits was found (*p*-value 0.0282); however, it was not possible to determine the correlation with the number of cigarettes smoked per day since some parameters had value 0 (e.g., in our sample, there were not non-smoking patients with a CPS value of more than 20). The majority of smokers (both heavy and light smokers) expressed a CPS higher than 1 (65.38%). No statistical correlation was detected between alcohol habit and CPS value (*p*-value 0.0612); also in this case, like for smoking, it was not possible to determine the correlation with the number of drinks drank per day (there were not light-drinkers in our sample). However, even for alcohol consumption as for smoking, most of drinkers had a CPS higher than 1 (75%). Elevated CRP levels (> 5 mg/L) were found in 26 patients (44.83%) - most of them expressed a CPS higher than 1 (73.08%); while 32 patients (55.17%) had CRP levels within the normal range. A statistically significant correlation was found between CPS value and this systemic inflammatory marker, confirming the role of PD-1/PD-L1 checkpoint in the inflammatory state typical of the LSCC (*p*-value 0.0014). Glottic cancers were the most common, affecting 27 patients (46.55%), followed by supraglottic (27.59%), extralaryngeal (13.80%), and transglottic (10.34%) cancers. Among patients affected by glottic cancer, 81.48% of them expressed a CPS value higher than 1. About primary tumor extension (T), our sample consisted of 10.34% of T1, 34.48% of T2, 37.93% of T3, 17.25% of T4. Except for T1, most of tumors (meant as primary tumor extension) had CPS higher than 1 and 20 equally. Only two cases were tumor recurrence. Most of patients didn’t have neck node involvement (N) (48.27%). Statistical analysis didn’t show a correlation between CPS value and T stage (*p*-value 0.5858); however, a statistically significant correlation with *N* stage was detected (*p*-value 0.0311). About the degree of differentiation, 75.86% of tumors were moderately differentiated (G2), 17.24% were poorly differentiated (G3), and 6.90% were well-differentiated (G1): the correlation between CPS and degree of differentiation didn’t result statistically significant. Moreover, CPS higher than 1 was found in 63.63% of G2 tumors (Tables 1 and 2). About CPS distribution, 65.52% of patients had a CPS ≥ 1, 17.24% had a CPS < 1, and 17.24% had a CPS > 20. The Spearman correlation matrix reveals significant associations between several variables and CPS, particularly with neck node involvement, T stage, and degree of differentiation. These correlations suggest a potential relationship between PD-L1 expression (CPS) and clinical parameters such as tumor progression and systemic inflammation (Table 3). The multivariate regression analysis identifies CRP (*p*-value 0.008), T stage (*p*-value 0.001), and neck node involvement (*p*-value 0.047) as significant predictors of PD-L1 expression (CPS) in patients with laryngeal squamous cell carcinoma. These findings suggest that systemic inflammation (CRP levels), tumor progression (T stage), and metastasis (neck node involvement) are key factors influencing PD-L1 expression (Table 4).

**Table 1:** Characteristics of enrolled patients (univariate analysis)

Characteristics	*N* (%)	Chi-squared test
**Sex**		
Females Males	18 (31.03) 40 (68.96)	0.0523
**Age (years)**		
Mean ± SD Range	63.55 ± 10.09 43–83	
**Smoking**		
Heavy-smokers Light-Smokers Non-smokers	46 (79.32) 6 (10.34) 6 (10.34)	**0.0282**
**Alcohol consumption**		
Heavy-drinkers Light-drinkers Non-drinkers	24 (41.38) 0 (0) 34 (58.62)	0.0612
**CRP (cut-off < 5 mg/L)**		
< 5 mg/L > 5 mg/L	32 (55.17) 26 (44.83)	**0.0014**
**Laryngeal subsite**		
Supraglottic Glottic Subglottic Transglottic Extended to pharynx	16 (27.59) 27 (46.55) 1 (1.72) 6 (10.34) 8 (13.80)	
**T (primary tumor extension)**		
T1 T2 T3 T4	6 (10.34) 20 (34.48) 22 (37.93) 10 (17.25)	0.5858
**Neck node involvement**		
Not known Not Yes	14 (24.14) 28 (48.27) 16 (27.59)	**0.0311**
**Degree of differentiation**		
G1 G2 G3	4 (6.90) 44 (75.86) 10 (17.24)	0.4501
**CPS**		
< 1 ≥ 1 > 20	10 (17.24) 38 (65.52) 10 (17.24)	
**Total**	**58 (100)**	

SD: standard deviation; CRP: C-reactive protein; CPS: combined positive score

**Table 2:** Distribution of CPS according to parameters

Parameters	CPS < 1	CPS ≥ 1	CPS > 20
**Sex**			
Females Males	4 6	8 30	6 4
**Age**			
Mean age ± SD Range	59.8 ± 2.99 56–65	62.78 ± 11.22 44–82	70.2 ± 6.49 65–83
**Smoking**			
Heavy-smokers Light-Smokers Non-smokers	8 0 2	28 6 4	10 0 0
**Alcohol consumption**			
Heavy-drinkers Light-drinkers Non-drinkers	4 0 6	18 0 20	2 0 8
**CRP (cut-off < 5 mg/L)**			
< 5 mg/L > 5 mg/L	4 6	18 20	10 0
**Laryngeal subsite**			
Supraglottic Glottic Subglottic Transglottic Extended to pharynx	2 4 2 0 2	14 14 0 4 6	0 8 0 2 0
**T (primary tumor extension)**			
T1 T2 T3 T4	2 2 4 2	2 14 16 6	2 14 16 6
**Neck node involvement**			
Not known Not Yes	0 4 6	14 14 10	0 10 0
**Degree of differentiation**			
G1 G2 G3	2 6 2	2 28 8	0 10 0
**Total**	**58**		

SD: standard deviation; CRP: C-reactive protein; CPS: combined positive score

**Table 3:** Spearman correlation matrix for the variables

Variable	Sex	Age	Smoking	Alcohol Consumption	CRP	Laryngeal Subsite	T Stage	Neck Node Involvement	Degree of Differentiation	CPS
Sex	1.000	NaN	-0.340	-0.798	-0.744	0.797	0.733	0.752	0.447	0.635
Age	NaN	NaN	NaN	NaN	NaN	NaN	NaN	NaN	NaN	NaN
Smoking	-0.340	NaN	1.000	0.426	0.457	-0.742	-0.691	-0.689	-0.810	-0.735
Alcohol Consumption	-0.798	NaN	0.426	1.000	0.932	-0.742	-0.860	-0.720	-0.470	-0.596
CRP	-0.744	NaN	0.457	0.932	1.000	-0.733	-0.909	-0.718	-0.482	-0.590
Laryngeal Subsite	0.797	NaN	-0.742	-0.742	-0.733	1.000	0.858	0.946	0.739	0.836
T Stage	0.733	NaN	-0.691	-0.860	-0.909	0.858	1.000	0.829	0.780	0.829
Neck Node Involvement	0.752	NaN	-0.689	-0.720	-0.718	0.946	0.829	1.000	0.694	0.817
Degree of Differentiation	0.447	NaN	-0.810	-0.470	-0.482	0.739	0.780	0.694	1.000	0.859
CPS	0.635	NaN	-0.735	-0.596	-0.590	0.836	0.829	0.817	0.859	1.000

**Table 4:** Multivariate Regression Analysis

Variable	Coefficient	Std. Error	t-value	*P*-value	95% Confidence Interval
Const	-1.2600	0.420	-3.003	0.004	[-2.103, -0.417]
Sex	0.1797	0.127	1.415	0.163	[-0.075, 0.435]
Smoking	-0.1127	0.118	-0.951	0.346	[-0.351, 0.125]
Alcohol consumption	7.073e-16	0.184	3.83e-15	1.000	[-0.371, 0.371]
CRP	0.6631	0.240	2.759	0.008	[0.180, 1.146]
Laryngeal subsite	-0.0560	0.089	-0.628	0.533	[-0.235, 0.123]
T stage	0.6251	0.174	3.591	0.001	[0.275, 0.975]
Neck node involvement	0.2512	0.123	2.042	0.047	[0.004, 0.498]
Degree of differentiation	0.0986	0.188	0.524	0.603	[-0.280, 0.477]

## Discussion

The immune system plays a key role against cancer. T-lymphocytes have an anti-tumor effect; however, their role is controlled by PD-1/PD-L1 axis: PD-1, expressed on surface of activated T-cells, binds to PD-L1, overexpressed in tumor cells, leading to T-cell inhibition and cancer spreading [5, 14]. PD-1 protein plays two opposite roles, as it can be beneficial and harmful. As for its positive effects, this protein plays a key role in containing the ineffective or even harmful immune response, promoting immune tolerance. However, PD-1 causes the growth and spread of cell carcinoma by interfering with the immune response against the tumor itself, thank to the immune tolerance mechanism [26]. Based on this finding, recent studies focused on researching drugs that could inhibit PD-1/PD-L1 checkpoint, promoting immune system response against tumor. So, as written before, the study KEYNOTE-048 demonstrated that pembrolizumab alone is more effective compared to cetuximab with chemotherapy (5-fluorouracil and platinum) and that pembrolizumab with chemotherapy ensures a better OS (*p*-value 0.0034) compared to cetuximab with chemotherapy [11]. Moreover, the best result of pembrolizumab alone was found in those patients with CPS ≥ 20 or ≥ 1.

In our study, we found a statistically significant correlation between CPS and blood CPR values, confirming the role and the activation of immune system in cancer. Franz et al. studied the correlation between PD-L1 CPS and blood neutrophils-to-lymphocytes ratio (NLR) (sign of immune response) funding a significant association between these parameters [13]. Like in our study, Franz et al. demonstrated the relationship between systemic inflammatory response and laryngeal immune microenvironment. However, the current literature focused on other systemic inflammatory markers such as NLR and platelet-to-lymphocyte ratio (PLR) [13]; on the contrary our research found a correlation between CPS and CPR values.

In their studies, Wang et al. and Kowalski et al. demonstrated a relationship between PD-1 expression level and TNM staging: PD-1 was overexpressed in advanced cancer (III and IV stages) with lymph node metastasis [14, 16]. Our study didn’t show any relationship between CPS and T stage; however, a statistically significant correlation was found between CPS and neck node involvement (*p*-value 0.0311). These authors also found an increased PD-L1 expression in glottic and subglottic LSCC (*p* < 0.05) [14]; in a similar way, our study showed that 81.48% of patients affected by glottic cancer expressed a CPS value higher than 1.

In their study, Yang et al. proved that PD-L1 expression level is higher in case of Human PapillomaVirus (HPV) positive LSCC samples and in non-smokers patients [15]. On the contrary, we demonstrated a statistically significant relationship between smoking habits and CPS expression levels. No correlation was found between alcohol assumption and CPS neither in literature nor in our research project.

About our sample, the majority of patients were males (68.96%) with mean age of 63.55 ± 10.09. Moreover, the most cases were glottic cancers (46.55%), followed by supraglottic ones (27.59%). These results were in accordance with the literature [27–29]. However, no correlation was found between CPS and these data.

Univariate analysis found a significant correlation between PD-L1 expression and smoking habits, CRP levels, and neck node involvement. Multivariate analysis confirmed the significant correlation between CPS and CRP and neck node involvement and found a correlation with T stage too. These findings support the involvement of the PD-1/PD-L1 checkpoint in the immune response to laryngeal carcinoma, particularly in the context of systemic inflammation and metastatic progression. If supported by a larger patient sample, these preliminary results suggest that PD-L1 expression could serve as a useful biomarker for tailoring immunotherapy in LSCC patients.

Two studies evaluated the possible correlation between PD-L1 and virus infections (Human Papillomavirus HPV and Epstein-Barr Virus EBV) [14, 30]. In particular, Yang et al. found that PD-L1 expression levels were statistically correlated with HPV infection (*p*-value = 0.001) [14]. Klatka et al. demonstrated that EBV infection may lead to expression of PD-1 receptors on T-lymphocytes, suggesting a better efficacy of immunotherapy in case of their combined presence [30].

The main strengths of this study are the prospective setting of the study (unlike other several studies [13, 17, 19, 20] and the homogeneity of the sample of patients. Indeed, 1) the surgical procedures were always performed by the same skilled surgeon; 2) all the patients were naïve, excluding cases of tumor recurrence or persistence; 3) only LSCC were included, not considering other Head and Neck sites, 4) the anti-PD-L1 mouse monoclonal clone 22C3 (22C3 IHC PharmDX) used is a commercial clone validated by experts, as well as the CPS score; 4) laboratory exams were performed by the same laboratory as well as the IHC assessment.

Anyway, the study has a main limitation that is the size of the sample. Indeed, we included only 58 patients in order to ensures the best homogeneity of enrolled sample. However, to date these results cannot be considered representative of the population affected by LSCC; indeed, these represent preliminary results and larger sample also with a multicentric analysis needs.

Moreover, we divided the patients in three groups based on CPS value. Other studies preferred a binary division on patients in CPS < 1 and CPS > 1. However, to date, studies proved that CPS > 20 does not mean eligibility for the first-line treatment with Keytruda as monotherapy. On the contrary, CPS ≥ 1 indicates the suitability of the patient for the first-line with treatment with Keytruda [31]. Moreover, some of tissue samples were achieved during the diagnostic procedure (biopsies) in transoral laryngeal microsurgery. So, both biopsies and specimen driven were assessed by IHC. Maybe, this may reduce the homogeneity of the included patients.

## Conclusion

This study investigates the correlation between PD-L1 expression levels (as measured by CPS) and various clinical parameters in patients affected by LSCC. The study found no correlation between PD-L1 expression levels and variables “sex”, “age”, “alcohol consumption”, “differentiation degree of LSCC”. Anyway, the study demonstrated the involvement of PD-1/PD-L1 checkpoint in the immune response in case of cancer since a relationship between CPS and CRP was found. A statistically significant correlation between PD-L1 expression levels and smoking habits and neck node status was found, although the small sample size suggests that clinically correlation is not as significant and further studies with more patients need. The significant correlations with smoking habits, CRP levels, and neck node involvement highlights the potential role of PD-L1 as a biomarker for tailoring immunotherapy in LSCC patients. These findings support the involvement of the PD-1/PD-L1 checkpoint in the immune response to laryngeal carcinoma, particularly in the context of systemic inflammation and metastatic progression. So, based on the poor literature, these results could pave the way for further studies with bigger samples that may help in the detection of the best patients tailored for treatment with anti-PD-1/PD-L1 blocking antibodies.
